# Assessment of Protective Effect of Some Modern Agrochemicals against Ozone-Induced Stress in Sensitive Clover and Tobacco Cultivars

**DOI:** 10.1155/2011/308598

**Published:** 2011-06-23

**Authors:** Oleg Blum, Nataliya Didyk, Nataliya Pavluchenko, Barbara Godzik

**Affiliations:** ^1^M. M. Gryshko National Botanical Garden, National Academy of Sciences of Ukraine, Timiryazevs'ka St., 1, 01014 Kyiv, Ukraine; ^2^W. Szafer Institute of Botany, Polish Academy of Sciences, Lubicz 46, 31-512 Krakow, Poland

## Abstract

Some modern agrochemicals with antioxidant potential were tested for their protective effect against ozone injury using clover and tobacco ozone-sensitive cultivars as model plants subjected to ambient ozone at two sites (Kyiv city in Ukraine and Szarów village in Poland). All used agrochemicals showed partial protective effects against ozone injury on clover and tobacco. Conducted studies confirmed the effectiveness of modern fungicides belonging to strobilurin group as protectants of sensitive crops against ozone damage. The effectiveness of new growth regulators “Emistym C” and “Agrostymulin” was showed for the first time. Out of the studied agrochemicals, fungicide “Strobi” and natural growth regulator “Emistym C” demonstrated the best protective effects. These agrochemicals present promise for further studies of their possible utilization for enhancement of ozone tolerance of sensitive crops.

## 1. Introduction

Ozone is one of the major toxic to vegetation and human health gaseous air pollutants. It contributes to crop losses and forest decline. It was estimated that the present day global relative yield losses range between 7% and 12% for wheat, between 6% and 16% for soybean, between 3% and 4% for rice, and between 3% and 5% for maize (range resulting from different metrics used) [1]. For the year 2000 global crop yield loss due to ambient ozone was estimated to be worth $14–26 billion. About 40% of this damage is occurring in China and India [1]. Crop yield loss due to ambient ozone in the U.S.A. was estimated to be worth $3–5 billion annually [2].

The ozone-induced crop losses could be mitigated by two ways: (i) selection of O_3_-tolerant cultivars; (ii) application of chemical protectants. So far there has been only limited success in producing genetically transformed plants with increased tolerance to elevated ozone concentrations because plant resistance to ozone is a very complicated phenomenon involving multiple signaling pathways and defense responses [3, 4].

The phytotoxicity of ozone results primarily from the oxidative stress imposed by the pollutant on sensitive components of the plasmalemma [3, 5]. In connection to this, application of antioxidants for protection of crops from ozone injury has been extensively studied over the last four decades [6, 7]. A large number of antioxidants (fungicides, insecticides, herbicides, growth regulators, etc.) have been evaluated [6]. Among them the systemic antioxidant ethylene diurea—N-[2-(2-oxo-1-imidazolidinyl) ethyl]-N′ phenylurea (EDU) was found to be the most efficient [6]. EDU, applied as a foliar spray, soil drench, or stem infusion agent, was shown to prevent acute ozone injury and inhibit plant senescence [6, 8]. Physiological effects of EDU associated with its protective properties are still unclear. Yet, there is some evidence that EDU may confer tolerance to ozone through the induction of enzyme systems involved in the elimination of activated oxygen species and free radicals [9].

Presently, modern agrochemicals containing antioxidant compounds, such as fungicides belonging to strobilurins and triazoles (azoxystrobin, epoxiconazole, penconazole, etc.), are gaining increasing attention as possible ozone protectants [10]. The above-mentioned fungicides, especially strobilurins, which were developed on the basis of natural substances extracted from the fungus Strobilurus tenacellus (Pers.) Singer, have low toxicity for human health and environment. Therefore they are more preferable for application in agriculture as protectants, than other synthetic agrochemicals such as EDU. In spite of the above-said a rather limited number of studies have been devoted to the protective effects of strobilurins against oxidative stress from the environment. Yet it was shown that application of strobilurins to plants caused marked enhancement of antioxidative enzymes and enhanced scavenging of potentially harmful reactive oxygen species.

Another promising group of agrochemicals is growth regulators containing phytohormones or/and others antioxidants. Apart from their effects on plant development, some phytohormones, that is, cytokinins, gibberellins, salicylic acid and their synthetic analogs were shown to enhance tolerance of crops to abiotic stresses including ambient ozone [6, 7]. The aim of this study was to assess protective properties of some modern agrochemicals with antioxidant potential including three fungicides belonging to strobilurins and some new growth regulators in comparison with EDU.

" 

## 2. Materials and Methods

The experiments were conducted simultaneously on two monitoring stations located in semiurban (National Botanic Garden in Kyiv city, Ukraine) and rural (Field Station of Institute of Botany Polish Academy of Sciences in Szarów, 30 km from Krakow, Poland) sites. In Kyiv plants of subterranean clover (Trifolium subterraneum L., O_3_-sensitive cv. Geraldton) and tobacco (Nicotiana tabacum L.) cvs. Bel-W3 (O_3_-sensitive) and Bel-B (O_3_-resistant) were used. In Szarów only subterranean clover was used. Prior to exposure to ozone, tobacco and clover were cultivated in ozone free air until they reach 4 leaves. In Kyiv plants were cultivated in the special chamber, made from organic glass, with the volume of 1.1 m^3^, at 25–28°C temperature, 65–70% RH, light illumination of 66 **μ**mol m^−2^·s^−1^ PAR and 14 : 10 (L : D) h photoperiod. The air was pumped up through charcoal filter with the rate of 0.3 m^3^ per min. In Szarów plants were cultivated in the special ozone free greenhouse at temperature of 25 ± 2°C with a relative humidity of 75% under natural photoperiod. Afterwards, the plants were sprayed with distilled water (control) or solutions of one of the following agrochemicals: (1) EDU (ethylenediurea), in concentrations of 150 mg L^−1^; (2) “Emistym C”, a natural growth regulator containing exometabolites of micorhizal fungi (flavonoids, phytohormones and organic acids), in concentration of 30 and 150 mg L^−1^; (3) “Agrostimulin,” containing synthetic growth regulator—ivin, in concentration of 30 and 150 mg L^−1^; modern fungicides belonging to the group of strobilurins such as (4) “Quadris” (containing 25% of azoxystrobin ) in concentration of 150 and 300 mg L^−1^; (5) “Flint” (containing 50% of trifloxystrobin) in concentration of 150 and 300 mg L^−1^; (6) “Strobi” (containing 50% of kresoxim-methyl) in concentration of 150 and 300 mg L^−1^. Ozone protective effect of “Emistym C” and “Agrostimulin” has been studied for the first time.

Two modes of agrochemical application (except for EDU) were studied. In Kyiv higher concentrations of the chemicals were applied only once (one day before exposure). In Sharów lower concentrations of agrochemicals were applied twice (one day before exposure and on 16th day of exposure). We used EDU as a standard ozone protectant, in concentration, whose effectiveness is well established on many plant species [6, 8].

The test plants were exposed into the field on the following day after application of agrochemicals. The duration of exposure of the test plants to ambient ozone was 2 and 4 weeks for tobacco and clover, respectively. On the last day of exposure ozone-induced foliar injury (%) and content of photosynthetic pigments (chlorophylls a and b, carotenoids) in leaves of test plants were evaluated. Photosynthetic pigments were extracted by 100% acetone and determined spectrophotometrically using “Spekol 11” (Carl zeiss, Jena, Germany) [11]. Dry biomass of test plants was also determined. For clover eight replications of ten plants each per treatment were used in both locations. Thus, values reported are the means of 80 plants. For tobacco ten replications of one plant each per treatment were used. Thus, values reported are the means of 10 plants.

During the period of the experiments ambient ozone concentrations were continuously monitored at the both locations. In Kyiv and Szarów ozone monitoring was conducted using the Thermo Environmental UV Photometric Ozone Analyzer Model 49-003 and Model 49 C, correspondingly. Average daily ozone and temperature concentrations as well as the doses of ozone, which test plants received during the period of experimentation were calculated using index of AOT 40 (accumulated exposure over a threshold of 40 ppb).

Statistical analysis of the results obtained was conducted with the usage of descriptive statistics and analysis of variance. Treatment means were compared using ANOVA and LSD-test (Statistica 6.0 software) [12].

## 3. Results and Discussion

**Figure 1:**
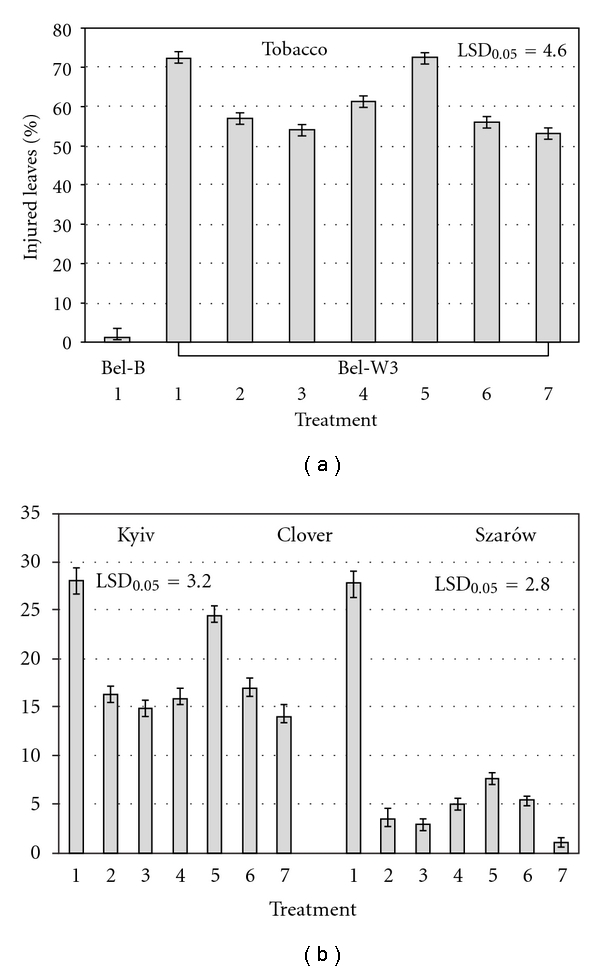
Ozone-induced visible injuries on leaves of clover and tobacco sprayed with distilled water (1), solutions of EDU, 150 mg L^−1^ (2), “Emistym C”, 150 mg L^−1^ in Kyiv and 30 mg L^−1^ in Szarów (3), “Agrostymulin”, 150 mg L^−1^ in Kyiv and 30 mg L^−1^ in Szarów (4), “Quadris”, 300 mg L^−1^ in Kyiv and 150 mg L^−1^ in Szarów (5), “Flint”, 300 mg L^−1^ in Kyiv and 150 mg L^−1^ in Szarów (6), “Strobi”, 300 mg L^−1^ in Kyiv and 150 mg L^−1^ in Szarów (7) on the last day of exposure to ambient ozone. Vertical bars represent standard error. LSD_0,05_: least significant difference at *P* < 0.05.

**Figure 2:**
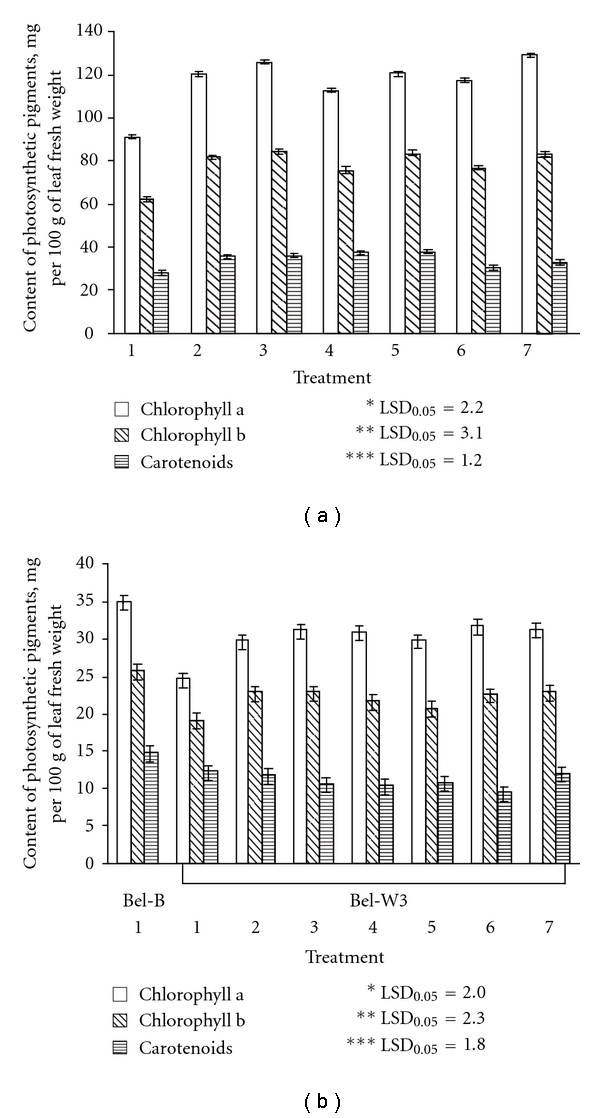
Content of photosynthetic pigments in leaves of clover (a) and tobacco (b) sprayed with distilled water (1), solutions of EDU, 150 mg L^−1^ (2), “Emistym C”, 150 mg L^−1^ (3), “Agrostymulin” 150 mg L^−1^ (4), “Quadris”, 300 mg L^−1^ (5), “Flint”, 300 mg L^−1^ (6), “Strobi”, 300 mg L^−1^ (7) on the last day of exposure to ambient ozone in Kyiv. Vertical bars represent standard error. LSD_0.05_: least significant difference at *P* < 0.05 for *chlorophyll a; **chlorophyll b; ***carotenoids.

**Figure 3:**
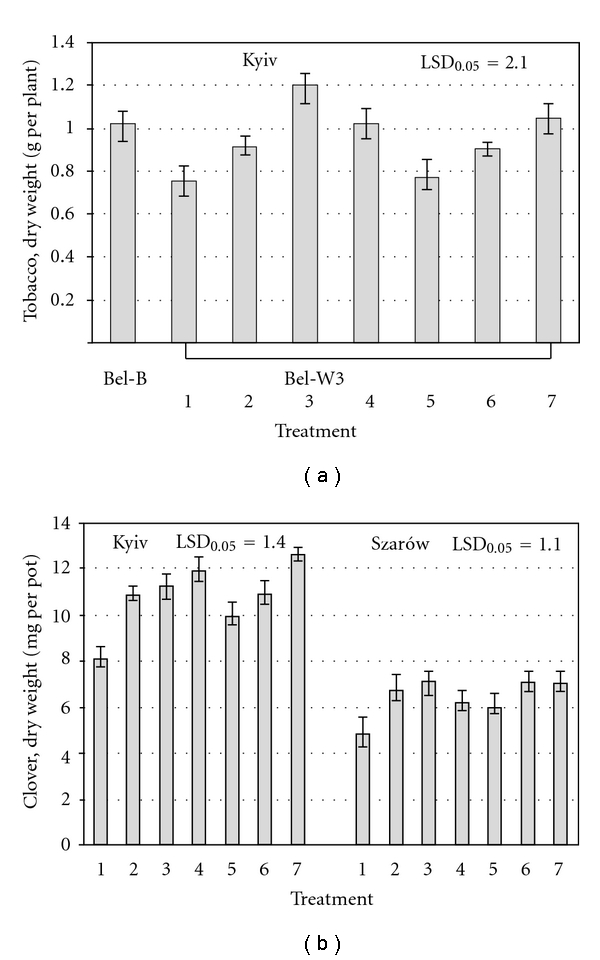
Mean dry weight of clover and tobacco sprayed with distilled water (control) (1), solutions of EDU, 150 mg L^−1^ (2), “Emistym C”, 150 mg L^−1^ in Kyiv and 30 mg L^−1^ in Szarów (3), “Agrostymulin”, 150 mg L^−1^ in Kyiv and 30 mg L^−1^ in Szarów (4), “Quadris”, 300 mg L^−1^ in Kyiv and 150 mg L^−1^ in Szarów (5), “Flint”, 300 mg L^−1^ in Kyiv and 150 mg L^−1^ in Szarów (6), “Strobi”, 300 mg L^−1^ in Kyiv and 150 mg L^−1^ in Szarów (7) on the last day of exposure to ambient ozone. Vertical bars represent standard error. LSD_0.05_— least significant difference at *P* < 0.05.

For the most part of the periods of observations the average daily ambient ozone concentrations exceeded the threshold of subterranean clover and tobacco Bel-W3 cv. sensitivity to ozone of 25 ppb [13, 14] in both experimental sites. The dose of ozone (calculated using index of AOT 40), which test plants received during the exposition periods, was 828 ppb·h for clover and 700 ppb·h for tobacco in Kyiv, and 1662 ppb·h for clover in Sharów. Despite the fact that in Szarów ambient ozone concentrations during the period of experimentation were higher than in Kyiv, clover plants in control (without application of agrochemicals) displayed similar degree of visible foliar injury (about 28%) in both sites (Figure 1). However, dry phytomass of clover plants in Sharów was almost two times lower than that in Kyiv. O_3_-sensitive tobacco plants demonstrated higher sensitivity to ozone than clover in terms of visible foliar injuries. All the applied agrochemicals (with the exception of “Quadris”, which have no significant effect on foliar injuries and biomass, accumulation in tobacco) showed partial protective effect to O_3_-sensitive plants. The plants treated with the solutions of the mentioned agrochemicals had less foliar injuries, higher biomass and higher content of photosynthetic pigments (chlorophylls a and b) in leaves as compared to control (sprayed with distil water) (Figures 1, 2, and 3). Tobacco plants treated with solutions of “Emistym C” had even higher biomass than plants of O_3_-resistant Bel-B cv. treated with distill water, which was evidently due to the growth promoting effect of this agrochemical rather than its ozone protective effect. Among the studied agrochemicals the most effective as protectant against ozone-induced injuries was fungicide “Strobi”. Natural growth regulator “Emistym C” was slightly less effective. Fungicide “Quadris” demonstrated the lowest effectiveness.

In terms of visible foliar injuries and biomass accumulation, all the applied agrochemicals more effectively protected clover plants when they were applied two times (one day before exposure and on 16th day of exposure) in lower concentration (in Szarów) than when they were applied once (one day before exposure) in higher concentration (in Kyiv). For biomass accumulation, this tendency was less defined. It could be supposed that repeated application of the tested agrochemicals even in lower concentrations is more effective as it allows protection of newly emerged leaves. Though, the differences in ozone doses, which plants received in both locations, could also contribute to the observed effects. The degree of protective effect for all tested agrochemicals was higher in clover plants as compared to tobacco. Evidently it was a consequence of higher degree of ozone injuries in tobacco as compared to clover.

Thus, conducted studies confirmed the effectiveness of ozone protective effect of EDU and fungicides belonging to the group of strobilurins [6, 8, 10]. The protective effect of growth regulators “Emistym C” and “Agrostimulin” against ozone damage of plants has been shown for the first time. Out of the studied agrochemicals fungicide “Strobi” and natural growth regulator “Emistym C” demonstrated the best protective effects. These agrochemicals present promise for further studies of their possible utilization for enhancement of ozone tolerance of sensitive crops.

## References

[1] Van Dingenen R, Dentener FJ, Raes F, Krol MC, Emberson L, Cofala J (2009). The global impact of ozone on agricultural crop yields under current and future air quality legislation. *Atmospheric Environment*.

[2] Fiscus EL, Booker FL, Burkey KO (2005). Crop responses to ozone: uptake, modes of action, carbon assimilation and partitioning. *Plant, Cell and Environment*.

[3] Chen Z, Gallie DR (2005). Increasing tolerance to ozone by elevating foliar ascorbic acid confers greater protection against ozone than increasing avoidance. *Plant Physiology*.

[4] Tosti N, Pasqualini S, Borgogni A (2006). Gene expression profiles of O_3_-treated Arabidopsis plants. *Plant, Cell and Environment*.

[5] Puckette MC, Tang Y, Mahalingam R (2008). Transcriptomic changes induced by acute ozone in resistant and sensitive Medicago truncatula accessions. *BMC Plant Biology*.

[6] Archambault D, Slaski DJ, Li JJ (2000). Ozone protection in plants. The potential use of chemical protectants to measure oxidant damage in Alberta crops. *Report prepared for the Air Research Users Group*.

[7] Didyk NP, Blum OB (2011). Natural antioxidants of plant origin against ozone damage of sensitive crops. *Acta Physiologiae Plantarum*.

[8] Godzik B, Manning WJ (1998). Relative effectiveness of ethylenediurea, and constituent amounts of urea and phenylurea in ethylenediurea, in prevention of ozone injury to tobacco. *Environmental Pollution*.

[9] Whitaker BD, Lee EH, Rowland RA (1990). EDU and ozone protection: foliar glycerolipids and steryl lipids in snapbean exposed to O_3_. *Physiologia Plantarum*.

[10] Wu YX, Von Tiedemann A (2002). Impact of fungicides on active oxygen species and antioxidant enzymes in spring barley (Hordeum vulgare L.) exposed to ozone. *Environmental Pollution*.

[11] Musiyenko MM, Parshikova TB, Slavnyi PC (2001). *Spectrophotometric Methods in the Practice of Physiology, Biochemistry and Ecology of Plants*.

[12] Zaytsev GN (1984). *Mathematical Statistics in Experimental Botany*.

[13] Karlsson GP, Pleijel H, Sild E (1995). Clover Sweden—a national three-year study of the effects of tropospheric ozone on Trifolium subterraneum, L.. *Water, Air, and Soil Pollution*.

[14] Heggestad HE (1991). Origin of Bel-W3, Bel-C and Bel-B tobacco varieties and their use as indicators of ozone. *Environmental Pollution*.

